# Ecophysiological Response of *Vitis vinifera* L. in an Urban Agrosystem: Preliminary Assessment of Genetic Variability

**DOI:** 10.3390/plants11223026

**Published:** 2022-11-09

**Authors:** Elena Brunori, Alessandra Bernardini, Federico Valerio Moresi, Fabio Attorre, Rita Biasi

**Affiliations:** 1 Department for Innovation in Biological, Agro-Food and Forestry Systems, University of Tuscia, 01100 Viterbo, Italy; 2 Department of Environmental Biology, Sapienza University of Rome, 00185 Rome, Italy

**Keywords:** agrobiodiversity, climate change adaptation, grapevine ecophysiology, spectral varietal signature, urban viticulture

## Abstract

Urban agriculture is an emerging challenge. Identifying suitable agrosystems that allow for the multiple functions of urban agriculture represents a key issue for the reinforcement of the agricultural matrix in cities, with the aims of counteracting and adapting to climate change and providing economic and social benefits. This study aims to produce a preliminary assessment of the adaptability of Italian native and non-native *Vitis vinifera* L. cultivars to the stressors of an urban environment. The investigation was carried out on the grapevine collection of the Botanical Garden of Rome (“Vigneto Italia”). A total of 15 grapevine varieties were selected for the evaluation of leaf chlorophyll content, stomatal conductance, and chlorophyll fluorescence under abiotic conditions during the growing season of 2021. Spectral signatures were collected from mature leaves, and several vegetation indices (LWI, MCARI, and WBI) were calculated. Our preliminary results highlighted differences in the behavior of the grapevine cultivars. The native ones showed a medium-high level for leaf chlorophyll content (greater than 350 mol m^−2^), good photosynthetic efficiency (QY > 0.75), and optimal stomatal behavior under drought stress (200 > gs > 50 mmol H_2_O m^−2^ s^−1^). The data allowed for the classification of the tested genotypes based on their site-specific resistance and resilience to urban environmental conditions. The grapevine proved to be a biological system that is highly sensitive to climate variables, yet highly adaptable to limiting growing factors.

## 1. Introduction

Urban agriculture (UA) contributes significantly to the objective of creating sustainable cities and attaining multiple sustainability goals (SDGs), as promoted by the UN Agenda 2030 [1] and by many other international initiatives, such as the European Green Deal and related strategies (Biodiversity, Farm to Fork Strategy, and the European Climate Pact) [2]. Although UA is widely recognized as a dispenser of various ecosystem services [3], it is pivotal to improve the knowledge of agrobiodiversity behavior in an urban environment, especially genotype–climate interaction, in order to maximize benefits and allow for healthier agronomical management [4,5]. Among urban agrosystems, vineyards [6] are gaining importance for their high-value production and cultural significance, in addition to their role as a crucial land-use for counteracting soil consumption and providing ecosystem services, such as carbon storage, biodiversity, and soil fertility [7,8,9,10,11,12]. 

Rome has been historically characterized by the presence of vineyards, which are, nowadays, highly fragmented and diminished due to urbanization and peri-urban agricultural-land abandonment [12]. The renewed interest in urban viticulture requires dealing with the ongoing climate changes [13] that endanger plant productivity and health. Abiotic conditions—atmosphere and soil variables—are crucial factors affecting grapevine ecophysiology and therefore the yields, the quality of berries, and vine health. Furthermore, *Vitis vinifera* L. is considered to be a bioindicator of climate changes, such as increased radiation, the frequency and severity of extreme weather events, the modification of precipitation patterns, on both a large [13,14] and a local scale [15], due its high susceptibility to light, thermal, and hydric fluctuations. 

Long-term adaptation strategies, adjusted to a local scale [16], which are useful for mitigating the negative impact of an urban microclimate on grapevine growth and grape quality, involve the selection of genotypes that better adapt to the multiple environmental stressors that characterize urban areas [17].

This study aimed to test the genotype (cultivar)-dependent ecophysiological behavior of the *Vitis vinifera* L. species grown in an urban environment, under common drought and hot microclimate conditions. This preliminary analysis involved 15 local, national, and international grapevine cultivars belonging to a collection of grapevine varieties cultivated in the Botanical Garden of Rome. The identification of genotypes that are more adaptable to an urban climate may allow for urban agricultural resilience by preserving local agrobiodiversity and typical production, and it is also crucial for urban ecological landscape design involving agrobiodiversity [18,19,20,21]. 

## 2. Results and Discussion

### 2.1. The Urban Environment

**Figure 1:**
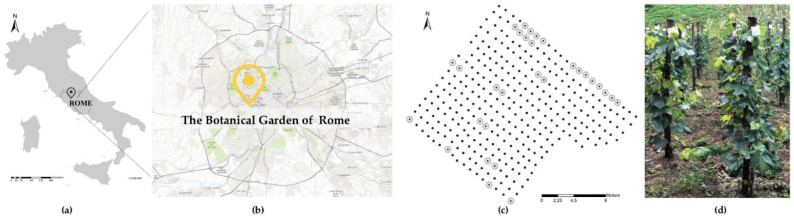
Geographical localization of the Botanical Garden in the center of Rome (**a**,**b**); map of the grapevine collection with the 15 selected genotypes (grey circles) (**c**); and vines trained to bush vine trellis systems (**d**).

Abiotic stressors in the urban vineyard of the Botanical Garden of Rome (Figure 1a,b) were monitored during the 2021 growing season. A Bagnouls—Gaussen climatic diagram (see Figure S1 in the Supplementary Materials) showed two dry periods obtained by comparing rainfall and average monthly temperature data from the meteorological station of the garden. A Mid-severe drought occurred during the flowering period (May), while a severe drought occurred from July to October. During the growing season, 213 dry days (from 1st April to 31 October 2021) were observed. There were 74 dry days between flowering (May) and berry set (July), and 41 dry days between berry ripening (August) and leaf fall (October). Furthermore, there were 69 days with a maximum temperature that exceeded 30 °C (June–August) and 18 days with a maximum temperature that exceeded 35 °C between the end of July and August (see Table 1).

Three water conditions were reported (Table 1): the mid-limited water condition (soil water content 36.4%, soil temperature 24.8 °C, and a medium dry spell) at BBCH 065 (full flowering), the unlimited water condition (soil water content 72%, soil temperature 26.2, and a short dry spell) and the limited water condition (soil water content 17.4–15%, soil temperature 31–32.1 °C, and a very long dry spell) at BBCH 089 (berries ripe for harvest) and 091 (after harvest–end of wood maturation), respectively. 

### 2.2. Physiological Responses of Grapevine Cultivars to Stressors

**Figure 2:**
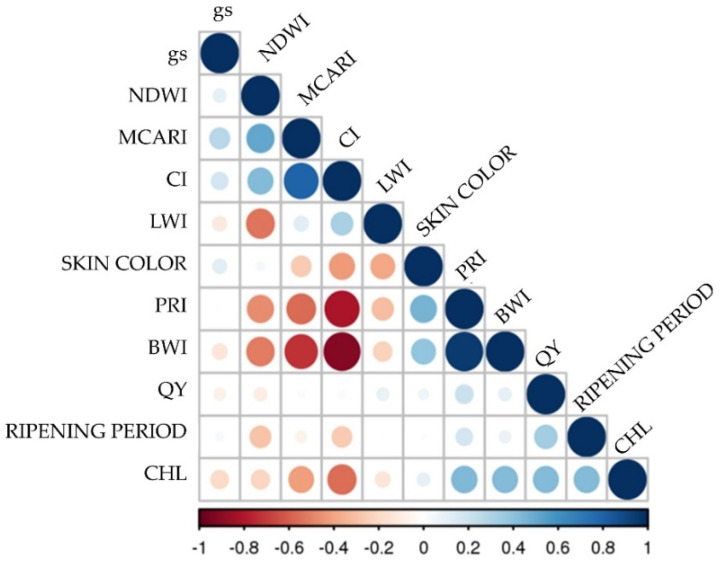
Correlogram (Pearson’s coefficient) of the hyperspectral indices, physiological and berry trait parameters for the 15 selected varieties. Positive correlations are displayed in blue and negative correlations in red. Circle diameter and color intensity are proportional to the correlation values as reported in the legend.

To assess the response of the selected cultivars (see Table 2) to the increasing level of drought and hot microclimate, we measured the leaf chlorophyll content, stomatal conductance, photosynthetic efficiency, and spectral signature (see Table 3). Correlation analysis (Pearson’s coefficient) was performed to evaluate the relationship among the spectral indices and the physiological parameters (see Figure 2). 

CHL exhibited good correlations with most physiological indicators. Specifically, it showed negative and significative correlations (Table S2) with CI (−0.55) and with the PRI and WBI (respectively, −0.82 and −0.91, Table S2). On the other hand, no significative correlations were found among QY, gs or the spectral indices. 

**Figure 3:**
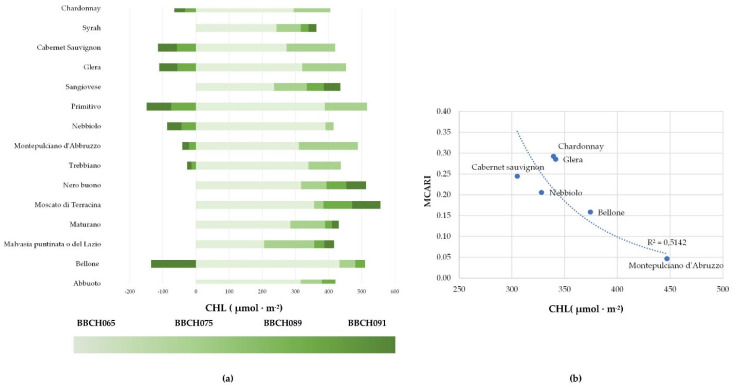
Trend of chlorophyll content (CHL) of selected grapevine cultivars during the 2021 growing season (**a**); Relationship between Modified Chlorophyll Absorption in the Reflectance Index (MCARI) and leaf chlorophyll content (**b**). Data refer to the measure at BBCH 091(after harvest–end of wood maturation).

During the growing season (April–October 2021) results exhibited genetic variability related to leaf senescence, as measured by leaf chlorophyll content, as reported by other authors [29,30]. A preliminary screening of the best performing grapevine cultivars demonstrated the rather high level of adaptability to stress conditions of many cultivars. While cvs. Chardonnay, Cabernet Sauvignon, Glera, Nebbiolo, Montepulciano, Bellone, Trebbiano, and Primitivo (Table 3, Figure 3), starting from BBCH 089 (berries ripe for harvest) to BBCH 091 (after harvest–end of wood maturation), showed a chlorophyll breakdown, other cultivars showed leaf CHL accumulation until the postharvest phenological stage. The CHL content in leaves of the cultivars that showed an early CHL breakdown (CHL, −100–150 μmol m^−2)^) resulted in correlations (Figure 3) to the MCARI, and it could therefore be elected as a proxy indicator of leaf senescence. Leaf senescence is a genetically controlled event in *Vitis vinifera* L., as in other deciduous trees [31], but it is also driven by abiotic and biotic factors [32].

It has been recognized that grapevine genotypes differ for stomatal behavior such that they have been classified as isohydric or anisohydric [33]. However, this classification may not be completely comprehensive and conclusive, and a genotype may show different stomatal behavior under different climatic, edaphic, and growth conditions [34,35,36]. Therefore, it becomes pivotal to understand the physiological performance of grapevine genotypes under local abiotic and biotic conditions as an ecological strategy for urban agrosystems. 

**Figure 4:**
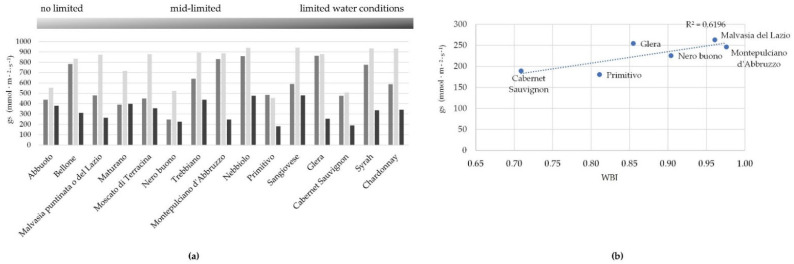
Stomatal conductance under different limited water conditions (**a**); relationship with water band index (WBI) for “water saving” genotypes (**b**). Data refer to the measure at BBCH 091 (after harvest–end of wood maturation).

In our study, stomatal behaviors were monitored by conductance (gs), as measured during the key phenological stages for grapevines. Based on the occurrence of abiotic stress (drought and a warm condition) and on gs values, some genotypes (Trebbiano, Nebbiolo, and Sangiovese) always showed high levels of gas exchanges (high values of conductance −700 > gs > 200 mmol H_2_O m^−2^ s^−1^) in mid-limited water conditions). The main ones reduced their stomatal conductance as drought advanced (limited water condition; see Figure 4). In particular, Primitivo, Cabernet Sauvignon, Nero buono, Montepulciano, Glera, and Malvasia del Lazio reflected this kind of “water saving” behavior. Local varieties have been proven to exhibit a higher WUE, based on net photosynthesis and a low gs [37], so they have better stomatal control under abiotic stress, closing stomata and reducing gas exchange. This last quality (gs) showed a linear correlation (Figure 4) with WBI, a proxy of plant water status [28]. Genotypic variation in stomatal behavior was confirmed in our study, as found by other authors [37], as a consequence of having a different leaf structure (e.g., stomata, leaf surface, and leaf size) [38]. 

**Figure 5:**
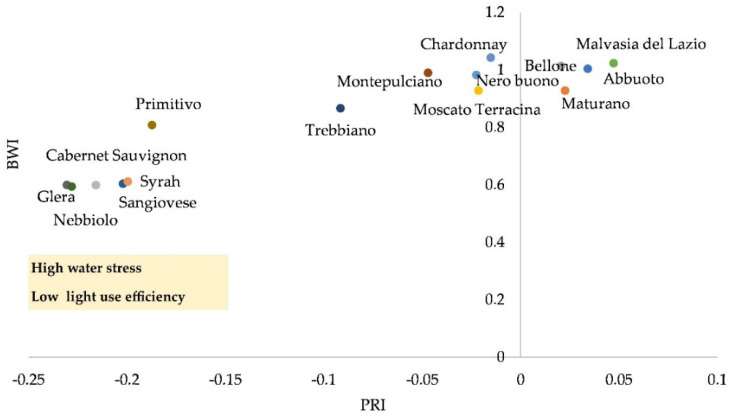
Water band index (WBI) and photochemical reflectance index (PRI): linear and significant correlation for 15 selected varieties. Data refer to the measure at BBCH 091 (after harvest–end of wood maturation).

Most cultivars (Table 3) exhibited a medium-high level of photosystem II efficiency (0.75 ≤ QY > 0.50 and QY > 0.75); only the local cultivar Bellone showed lower efficiency under extended drought (QY 0.57). Although some cultivars exhibited an early CHL breakdown, and/or a lower level of QY, some showed good light-use efficiency, preserving water content in vines (Figure 5), therefore providing an indication of the efficiency of carbon storage in their perennial organs. 

### 2.3. Hyperspectral Genotype Signature

**Figure 6:**
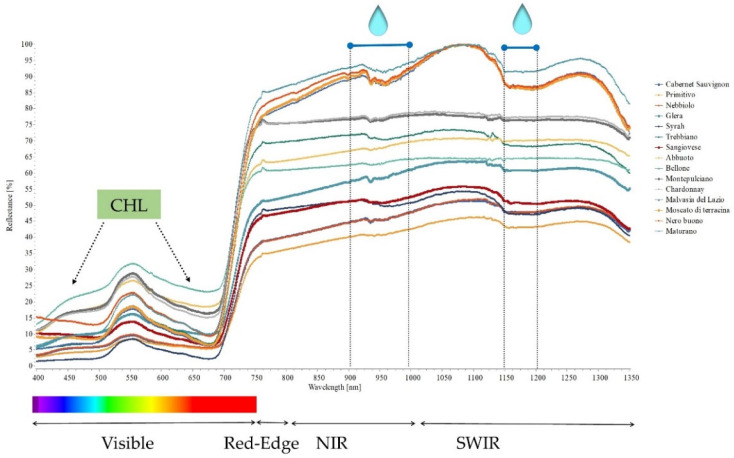
Hyperspectral signature of the tested cultivars. Measurements refer to the highest stress conditions, BBCH 089 (berries ripe for harvest).

Hyperspectral measurement can represent an indirect method for assessing many plant responses. The hyperspectral genotype signature (Figure 6), from the visible to the near-infrared region (NIR) and a part of the short-wave infrared (SWIR) wavelengths, exhibited the sensitivity of leaf water content [39,40] and confirmed the highly variable behavior of grapevines. In particular, in our study, all vines exhibited a similar trend for the reflectance spectra, showing three distinctive features: a peak at 540–560 nm, a trough at 680–690 nm, and a plateau in the near-infrared region above approximately 770 nm. Findings confirmed that all spectra were peculiar for drought conditions, and the vines differed in the water absorption band located within the NIR, around 980 nm, and in the second domain (SWIR; Figure 6) related to leaf traits [39], which is also affected by water content. 

Hyperspectral signatures provide useful information on the behavior of the cultivars under stress conditions. Spectral assessment is not very time-consuming although it needs to be calibrated to the main physiological parameters [38,41] or to metabolic markers [42], giving useful information for a rapid selection of cultivars that are better adapted to environmental stressors or for breeding programs. Our results highlight the potential of using spectral measurements as an indicator of competition for water. Taken together, the findings prove the importance of local knowledge, not only for choosing the right cultivar, but also for defining agronomical adaptation strategies [43,44].

**Figure 7:**
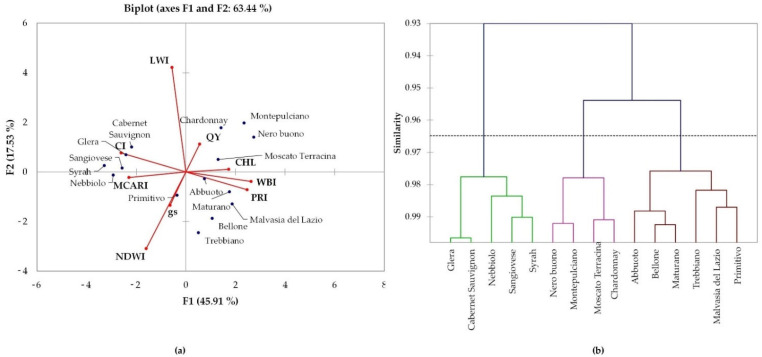
Principal component analysis (PCA) (**a**) and dendrogram of agglomerative hierarchical clustering (AHC) (**b**) obtained using physiological data for the 15 selected genotypes in the metropolitan area of Rome.

Finally, statistical AHC (Figure 7) allowed us to categorize genotypes based on physiological response to abiotic stressors in an urban area. In general, the local cultivars (those of Central Italy) were positively related to leaf chlorophyll content, light use, and photosynthetic efficiency. Our results may represent a preliminary classification of suitable grapevine genotypes that are better adapted to the microclimate growing area of metropolitan Rome.

## 3. Materials and Methods

### 3.1. Study Site and Plant Material

A vineyard was planted in 2018 within the Botanical Garden of Rome (Figure 1a,b). It represents a core collection (named the “Italian vineyard”) of 154 Italian and international grapevine cultivars, each one represented by two vines grafted onto V. *berlandieri* × *rupestris* or V. *berlandieri* × *riparia*, managed according to the principles of organic farming and trained to a bush vine trellis system (vertical shoot positioning; planting distance: 1 m × 0.5 m) (Figure 1c,d). During the 2021 season, it was monitored for the ecophysiological response to urban pedo-climatic conditions of 12 autochthonous grapevine varieties, chosen among those in the collection as being representative of different geographical provenances (North, Central, and Southern Italy), for berry skin color (white and black) and ripening time (early, medium, and late ripening). Three international varieties (cvs Chardonnay, Cabernet Sauvignon, and Syrah) were also included in the study (Table 2). The selected cultivars were representative of the three clusters identified by a larger, preliminary germplasm comparison of ecophysiological response to an urban environment [8].

Meteorological data (daily average, maximum, and minimum temperature; and amount of precipitation) were obtained for the 2021 season in continuum by a weather station (Olinda TECNO.EL) located in the Botanical Garden of Rome. Average monthly precipitation and average monthly temperature were used to elaborate ombrothermic diagrams [45]. Meteorological data were used to determinate extreme events [15] related to rainfall and temperature regime, in particular: (i) the number of consecutively dry days with no precipitation or with precipitation below the threshold of 1 mm; (ii) the number of days in which the maximum temperature exceeded 30 °C and 35 °C. Furthermore, soil temperature and moisture were assessed by a FieldScout TDR 350 soil moisture meter (Spectrum Technologies, Inc., Aurora, IL, USA) any time the physiological determinations were carried out. The values of the selected climatic indices were measured at the same time that the physiological determinations occurred, allowing for the identification, for each sampling datum, of three local environmental categories: unlimited, mid-limited, and limited water conditions (Table 1).

### 3.2. Ecophysiological Determinations

Data were collected at key phenological phases for grapevines (i.e., full flowering, fruit set, berry growth, berry ripening, and end of wood maturation) according to BBCH classifications [8]. Physiological determinations conducted on two vines per cultivar consisted of the measurement of the stomatal conductance (gs-mmol H_2_O m^−2^·s^−1^) as tested on three fully expanded undamaged sunlit leaves (the second leaf above the last bunch of each main shoot) between 10:30 a.m. and 12:30 p.m. [46] The stomatal conductance was assessed near the central vein of the leaf blade [47] with a leaf porometer (SC-1, Decagon Devices, Pullman, WA, USA). The same leaves were used to quantify: (i) the chlorophyll leaf content (CHL—μmol m^−2^) as a leaf-senescence indicator [48] using a hand-held MC-100 Chlorophyll Concentration Meter (Apogee Instruments, Inc., Logan, UT, USA); (ii) the quantum yield of photosystem II (QY-mol·mol^−1^ quanta absorbed) recorded by the portable fluorescence meter, FluorPen FP 110 (Photon systems instruments, Czech Republic), which also allowed for the measurement of photosynthetically active radiation (400 to 700 nm—PAR–PPFD). Two measurements for each photochemical parameter were made per leaf, per tested vine. 

The diffuse reflectance spectra of each tested cultivar were also detected using a portable, high-resolution spectrophotometer (ASD FieldSpec^®^3, Analytical Spectral Devices, Boulder, CO, USA) that recorded the full range of the solar irradiance spectrum (350–2500 nm). A total of 15 hyperspectral measurements were made on each leaf used for the physiological measurements, according to the following phenological stages: BBCH 065 (full flowering), BBCH 079 (majority of berries touching), BBCH 089 (berries ripe for harvest), and BBCH 091(after harvest–end of wood maturation). A reflectance spectral signature was derived for each tested grapevine variety by averaging the data from 15 replicates. 

Finally, based on reflectance at specific wavelengths, vegetation indices were calculated. The indices were selected for their usefulness in assessing the vines’ physiological performance under multiple abiotic stressors, such as high temperatures and drought in accordance with other authors [28,38,49]:

PRI = R530 − R550/R530 + R550

The photochemical reflectance index measures light-use efficiency, providing an indication of the efficiency with which vegetation uses incident light for photosynthesis;

MCARI = [(R700/R670) − 0.2 ∗ (R700/R550)] ∗ (R700/R670)

The modified chlorophyll absorption in reflectance index, simplified by Daughtry et al., (2000), is an alternative index that provides a measure of chlorophyll concentration, but it is also sensitive to non-photosynthetic components of the canopy, especially at low chlorophyll concentrations;

CI = (R750/R718RE0) − 1

The Chlorophyll Index - Red Edge is correlated with chlorophyll content and should be a good proxy for the phenology of crops;

WBI = R950/R900

The Water Band Index is a canopy-water content index which has been proven to track the changes in the relative water content of crops;

NDWI = (R857 − R1241)/(R857 + R1241)

The normalized difference water index is related to the photosynthetically active radiation absorbed by the greener and healthier leaves. Therefore, it is considered to be an implement for interpreting the spatial patterns of the canopy, such as changes in canopy size, photosynthetic capacity, and canopy chlorophyll content; structure and plant health status; crop productivity; nutrient or water stress; and berry characteristics, which can be related to changes in the microclimate or other conditions:

LWI = R1300/R1450

The leaf water index is used as a representation of water content in vegetation. Plants that have decreased health will experience a decrease in water content.

### 3.3. Statistics

Statistical analysis was performed with respect to the data matrix consisting of the averaged values determined for all of the previously described variables. The Pearson correlation test was used to determine the relationship between the measured physiological parameters (gs, QY, and CHL) and the vegetational indices derived from spectrophotometric measurements (PRI, MCARI, CI, WBI, NDWI, and LWI). Two-way ANOVA analysis was also carried out to study the effect of berry skin color, as well as their ripening period, on the vegetational indices (MCARI, PRI, CI, WBI, LWI, and NDWI) and on their physiological parameters (QY, gs, and CHL). 

Agglomerative hierarchical clustering (AHC) was carried out using Pearson’s correlation coefficient and complete linkage as the agglomeration method so as to obtain a similarity matrix and a dendrogram of the clusters. Principal component analysis (PCA) was performed to assess the relationship between the ecophysiological parameters and the vegetational indices. All data were analyzed using XLSTAT software (Addinsoft, www.xlstat.com accessed on 1 July 2022).

## 4. Conclusions

The world’s population will increasingly concentrate in metropolitan areas, and cities will also increasingly become places of primary food production. To allow cities to become inclusive, safe, resilient, and sustainable, starting from more-sustainable food systems is a key issue that drives environmental, social, and economic strategies and actions. Enhancing the potential of UA requires an integrated and ecosystems-based approach that considers: (i) mitigation strategies, e.g., short-term actions to reduce greenhouse gas emissions based on agricultural practices; and (ii) adaptation strategies, e.g., long-term actions reducing the vulnerability to climate change by cultivating a better-adapted agrobiodiversity. The success of future UA in semi-arid areas, such as Mediterranean cities, requires the identification of drought- and heat-tolerant varieties so as to reduce the ecological impact of the agronomical practices and to increase environmental benefits. 

Our findings highlight the different potential resistances to multiple abiotic stressors of some of the main grapevine varieties, in particular: the different capacities for maintaining better light-use efficiency and for regulating the leaf water status, thereby providing supporting and regulating environmental benefits (mainly carbon sequestration and storage). Local varieties showed a great level of stomatal control that allows for increasing photosystem II efficiency and delayed CHL breakdown under both progressive drought and hot microclimate conditions. Mediterranean wine-grape growing regions can already benefit from a wide range of varietal choices. Nonetheless, it is a challenge to find new varieties with improved resistance to future warmer and drier climates. This study showed that the co-evolution of terroirs and local grapevine landraces will allow them to be more susceptible for the bio-cities of the future. 

## Figures and Tables

**Table 1:** Definition of water conditions (unlimited, mid-limited, and limited) according to abiotic parameters: dry spell duration (number of consecutive dry days), soil temperature (°C), and soil water content (%).

	Water Condition			
	Unlimited	Mid-Limited	Limited	Reference
Dry Spell Duration	Very short to short	Medium	Long to very long	[22]
dry days (n)	7 ÷ 13	14 ÷ 19	>20	
Soil temperature				[23]
(°C)	<22	22 ÷ 30	>30	
Soil water content	Saturated	Medium	Low	[24]
(%)	>70	20 ÷ 70	<20	

**Table 2:** Selected grapevine varieties grouped by geographical provenience, berry color, and ripening period.

Growing Area	Cultivar	Berry Color/Ripening Period ^1^
Northern Italy		
Piemonte	Nebbiolo	B/E
Veneto	Glera	W/L
Central Italy		
Lazio	Abbuoto	B/L
	Malvasia del Lazio	W/L
	Moscato Terracina	W/M
	Bellone	W/M
	Maturano	W/M
	Nero buono	B/M
Abbruzzo	Trebbiano	W/M
	Montepulciano	B/M
Toscana	Sangiovese	B/L
Southern Italy		
Puglia	Primitivo	B/E
International varieties	Chardonnay	W/E
	Syrah	B/M
	Cabernet Sauvignon	B/L

^1^ W: white berry, B: black berry; E: early, M: medium, L: late ripening period.

**Table 3:** Summary of key results categorizing cultivars according to leaf pigments (chlorophyll content), stomatal behavior under period of drought, and photochemical efficiency of PSII (quantum yield: QY). Data refer to the measure at BBCH 091 (after harvest–end of wood maturation).

	Category			
Physiological Criterion	High	Medium	Low	Reference
**Leaf pigments**	[CHL] > 350 mol m^−2^	350 μmol · m^−2^ ≤ [CHL] > 250 μmol m^−2^	[CHL] ≤ 250 μmol m^−2^	[25]
Leaf chlorophyll content (CHL)	Malvasia del Lazio, Maturano, Moscato di Terracina, Nero buono, Trebbiano, Sangiovese	Syrah	Chardonnay, Cabernet Sauvignon, Glera, Nebbiolo, Montepulciano, Bellone, Primitivo, Abbuoto	
**Stomatal behavior under drought stress**	700 > gs > 200 mmol H_2_O m^−2^ s^−1^	200 > gs > 50 mmol H_2_O m^−2^ s^−1^	gs ≤ 50 mmol H_2_O m^−2^ s^−1^	[26]
Stomatal conductance (gs)	Trebbiano, Nebbiolo, Sangiovese	Syrah, Chardonnay, Moscato di Terracina, Abbuoto, Maturano	Primitivo, Cabernet Sauvignon, Nero buono, Montepulciano, Glera, Malvasia del Lazio	
**Functionality of photosynthetic apparatus**	QY > 0.75	0.75 ≤ QY > 0.50	QY ≤ 0.50	[27]
Photosynthetic activity (QY)	Glera, Chardonnay, Abbuoto, Cabernet Sauvignon, Nero buono, Sangiovese, Maturano, Malvasia del Lazio	Nebbiolo, Primitivo, Syrah, Moscato di Terracina, Trebbiano, Montepulciano	Bellone	
Photochemical reflectance index (PRI)	Montepulciano, Maturano, Malvasia del Lazio, Glera, Syrah	Abbuoto, Primitivo, Sangiovese, Nebbiolo, Moscato di Terracina, Nero buono, Trebbiano, Cabernet Sauvignon	Bellone, Cabernet Sauvignon	[28]

## Data Availability

Not applicable.

## Supplementary Materials

The following supporting information can be downloaded at: https://www.mdpi.com/article/10.3390/plants11223026/s1, Figure S1: Climatic Bagnouls and Gaussen diagram and dry periods (red area) for the season 2021 in urban vineyard of Botanical Garden of Rome; Table S1: Two way ANOVA performed to study the effect of berry skin color and as well as ripening period on vegetational indices (MCARI, PRI, CI, WBI, LWI, NDWI) and physiological parameters (QY, gs and CHL) of leaves of 15 selected varieties at BBCH 089- Berries ripe for harvest.

## References

[1] Department of Economic and Social Affairs, United Nation (2016). Transforming Our World: The 2030 Agenda for Sustainable Development.

[2] European Commission A European Green Deal. https://ec.europa.eu/info/strategy/priorities-2019-2024/european-green-deal_en.

[3] Clinton N., Stuhlmacher M., Miles A., Uludere N., Wagner M., Georgescu M., Herwig C., Gong P. (2018). A global geospatial ecosystem services estimate of urban agriculture. Earth’s Future.

[4] FAO, Rikolto, RUAF (2022). Urban and PERI-Urban Agriculture Sourcebook–From Production to Food Systems.

[5] Singh R.P. (2018). Integration and commercialization of local varieties under sub-optimal environments for food security, promoting sustainable agriculture and agrobiodiversity conservation. MOJ Ecol. Environ. Sci..

[6] OIV Statistical Report on World Vitiviniculture. http://www.oiv.int/public/medias/6782/oiv-2019-statistical-report-on-world-vitiviniculture.pdf.

[7] Biasi R., Barbera G., Marino E., Brunori E., Nieddu G. Viticulture as crucial cropping system for counteracting the desertification of coastal land. Proceedings of the XXVIII International Horticultural Congress on Science and Horticulture for People: International Symposium on the Effect of Climate Change on Production and Quality of Grapevines and their Products.

[8] Biasi R., Brunori E., Moresi F.V., Maesano M., Cipriani F., Carpentieri S., Rossetti L., Attorre F. (2022). Resilience and resistance of viticultural biodiversity in the urban ecosystem: The case of the grapevine collection of the Botanical Garden of Rome. Acta Hortic..

[9] Brunori E., Farina R., Biasi R. (2016). Sustainable viticulture: The carbon-sink function of the vineyard agro-ecosystem. Agric. Ecosyst. Environ..

[10] Candiago S., Winkler K.J., Giombini V., Giupponi C., Vigl L.E. (2022). An ecosystem service approach to the study of vineyard landscapes in the context of climate change: A review. Sustain. Sci..

[11] Garcia L., Celette F., Gary C., Ripochen A., Valdés-Gómez H., Metay A. (2018). Management of service crops for the provision of ecosystem services in vineyards: A review. Agric. Ecosyst. Environ..

[12] Brunori E., Salvati L., Mancinelli R., Smiraglia D., Biasi R. (2017). Multi-temporal land use and cover changing analysis: The environmental impact in Mediterranean area. Int. J. Sustain. Dev. World Ecol..

[13] Hannah L., Roehrdanz P.R., Ikegami M., Shepard A.V., Shaw M.R., Tabor G., Zhi L., Marquet P.A., Hijmans R.J. (2013). Climate change, wine, and conservation. Biol. Sci..

[14] Jones G.V., Davis R.E. (2000). Climate influences on grapevine phenology, grape composition, and wine production and quality for Bordeaux, France. Am. J. Enol. Vitic..

[15] Biasi R., Brunori E., Ferrara C., Salvati L. (2019). Assessing impacts of climate change on phenology and quality traits of *Vitis vinifera* L.: The contribution of local knowledge. Plants.

[16] Santos J.A., Yang C., Fraga H., Malheiro A.C., Moutinho-Pereira J., Dinis L.T., Correia C., Moriondo M., Bindi M., Leolini L. (2021). Long-Term Adaptation of European Viticulture to Climate Change: An Overview from the H2020 Clim4Vitis Action.

[17] Hunter J.J.K., Tarricone L., Volschenk C., Giacalone C., Susete Melo M., Zorer R. (2020). Grapevine physiological response to row orientation-induced spatial radiation and microclimate changes. OENO One.

[18] De la Salle J., Holland M. (2010). Agricultural Urbanism: Handbook for Building Sustainable Food & Agriculture Systems in 21st Century Cities.

[19] Duru M., Therond O., Martin G., Martin-Clouaire R., Magne M.A., Justes E., Sarthou J.P. (2015). How to implement biodiversity-based agriculture to enhance ecosystem services: A review. Agron. Sustain. Dev..

[20] Ibañez D., Guallart V., Salka M. (2022). On pedagogical prototyping of advanced ecological buildings and biocities at Valldaura Labs. AGATHÓN Int. J. Archit. Art Des..

[21] Viljoen A., Bohn K., Howe J. (2005). Continuous Productive Urban Landscapes: Designing Urban Agriculture for Sustainable Cities.

[22] Tabari H., Mendoza Paz S., Buekenhout D., Willems P. (2021). Comparison of statistical downscaling methods for climate change impact analysis on precipitation-driven drought. Hydrol. Earth Syst. Sci..

[23] Pogačar T., Zupanc V., Kajfež Bogataj L., Črepinšek Z. (2018). Soil temperature analysis for various locations in Slovenia. Ital. J. Agrometeorol..

[24] Wei J., Li X., Liu L., Røjle Christensen T., Jiang Z., Ma Y., Wu X., Yao H., López-Blanco E. (2022). Radiation, soil water content, and temperature effects on carbon cycling in an alpine swamp meadow of the northeastern Qinghai–Tibetan Plateau. Biogeosciences.

[25] Steele M., Gitelson A.A., Rundquist D. (2008). Nondestructive estimation of leaf chlorophyll content in grapes. Am. J. Enol. Vitic..

[26] Cifre J., Bota J., Escalona J.M., Medrano H., Flexas J. (2005). Physiological tools for irrigation scheduling in grapevine (*Vitis vinifera* L.) An open gate to improve water-use efficiency?. Agric. Ecosyst. Environ..

[27] Guidi L., Lo Piccolo E., Landi M. (2019). Chlorophyll fluorescence, photoinhibition and abiotic stress: Does it make any difference the fact to be a C3 or C4 species?. Front. Plant Sci..

[28] Sellami M.H., Albrizio R., Colovíc M., Hamze M., Cantore V., Ťodorovic M., Piscitelli L., Stellacci A.M. (2022). Selection of hyperspectral vegetation indices for monitoring yield and physiological response in sweet maize under different water and nitrogen availability. Agronomy.

[29] Riffle V., Palmer N., Casassa L.F., Dodson Peterson J.C. (2021). The effect of grapevine age (*Vitis vinifera* L. cv. Zinfandel) on phenology and gas exchange parameters over consecutive growing seasons. Plants.

[30] Cataldo E., Salvi L., Sbraci S., Storchi P., Mattii G.B. (2020). Sustainable Viticulture: Effects of Soil Management in *Vitis vinifera*. Agronomy.

[31] Mattila H., Valev D., Havurinne V., Khorobrykh S., Virtanen O., Antinluoma M., Mishra K.B., Tyystjärvi E. (2018). Degradation of chlorophyll and synthesis of flavonols during autumn senescence—The story told by individual leaves. AoB Plants.

[32] Hörtensteiner S., Kräutler B. (2011). Chlorophyll breakdown in higher plants. Biochim. Biophys. Acta.

[33] Palliotti A., Tombesi S., Frioni T., Silvestroni O., Lanari V., D’Onofrio C., Matarese F., Bellincontro A., Poni S. (2015). Physiological parameters and protective energy dissipation mechanisms expressed in the leaves of two *Vitis vinifera L.* genotypes under multiple summer stresses. J. Plant Physiol..

[34] Lovisolo C., Perrone I., Carra A., Ferrandino A., Flexas J., Medrano H., Schubert A. (2010). Drought-induced changes in development and function of grapevine (*Vitis* spp.) organs and in their hydraulic and non-hydraulic interactions at the whole-plant level: A physiological and molecular update. Funct. Plant Biol..

[35] Collins M.J., Fuentes S., Barlow E.W.R. (2010). Partial rootzone drying and deficit irrigation increase stomatal sensitivity to vapour pressure deficit in anisohydric grapevines. Funct. Plant Biol..

[36] Bianchi D., Grossi D., Tincani D.T.G., Di Lorenzo G.S., Brancadoro L., Rustioni L. (2018). Multi-parameter characterization of water stress tolerance in *Vitis* hybrids for new rootstock selection. Plant Physiol. Biochem..

[37] Bota J.M., Tomás J., Flexas J., Medrano H., Escalona J.M. (2016). Differences among grapevine cultivars in their stomatal behavior and water use efficiency under progressive water stress. Agric. Water Manag..

[38] Cogato A., Wu L., Jewan S.Y.Y., Meggio F., Marinello F., Sozzi M., Pagay V. (2021). Evaluating the spectral and physiological responses of grapevines (*Vitis vinifera* L.) to heat and water stresses under different vineyard cooling and irrigation strategies. Agronomy.

[39] Laroche-Pinel E., Albughdadi M., Duthoit S., Chéret V., Rousseau J., Clenet H. (2021). Understanding vine hyperspectral signature through different irrigation plans: A first step to monitor vineyard water status. Remote Sens..

[40] Imanishi J., Sugimoto K., Morimoto Y. (2004). Detecting drought status and LAI of two *Quercus* species canopies using derivative spectra. Comput. Electron. Agric..

[41] Ronay I., Ephrath J.E., Eizenberg H., Blumberg D.G., Maman S. (2021). Hyperspectral reflectance and indices for characterizing the dynamics of crop–weed competition for water. Remote Sens..

[42] Florez-Sarasa I., Clemente-Moreno M.J., Cifre J., Capó M., Llompart M., Fernie A.R., Bota J. (2020). Differences in metabolic and physiological responses between local and widespread grapevine cultivars under water deficit stress. Agronomy.

[43] Anderson R., Bayer P.E., Edwards D. (2020). Climate change and the need for agricultural adaptation. Curr. Opin. Plant Biol..

[44] Del Pozo A., Brunel-Saldias N., Engler A., Ortega-Farias S., Acevedo-Opazo C., Lobos G.A., Jara-Rojas R., Molina-Montenegro M.A. (2019). Climate change impacts and adaptation strategies of agriculture in Mediterranean-Climate Regions (MCRs). Sustainability.

[45] Jones G.V., Macqueen R.W., Meinert L.D. (2007). Climate and terroir: Impacts of climate variability and change on wine. Fine Wine and Terroir—The Geoscience Perspective.

[46] Johnson D.M., Woodruff D.R., McCulloh K.A., Meinzer F.C. (2009). Leaf hydraulic conductance, measured in situ, declines and recovers daily: Leaf hydraulics, water potential and stomatal conductance in four temperate and three tropical tree species. Tree Physiol..

[47] Stavrinides M.C., Daane K.M., Lampinen B.D., Mills N.J. (2010). Plant water stress, leaf temperature, and spider mite (Acari: Tetranychidae) outbreaks in California vineyards. Environ. Entomol..

[48] Cavallo P., Poni S., Rotundo A. (2001). Ecophysiology and vine performance of cv. “Aglianico” under various training systems. Sci. Hortic..

[49] Darra N., Psomiadis E., Kasimati A., Anastasiou A., Anastasiou E., Fountas S. (2021). Remote and proximal sensing-derived spectral indices and biophysical variables for spatial variation determination in vineyards. Agronomy.

