# Supplementary material for: Ecophysiological Response of Vitis vinifera L. in an Urban Agrosystem: Preliminary Assessment of Genetic Variability
Source: Plants (Basel). 2022 Nov 9;11(22):3026. doi: 10.3390/plants11223026 (PMC9694217; doi:10.3390/plants11223026)
Supplement: Supplementary file 1 [file plants-11-03026-s001.zip › plants-1980833-supplementary.pdf]

plants

MDPI

Supplementary Materials

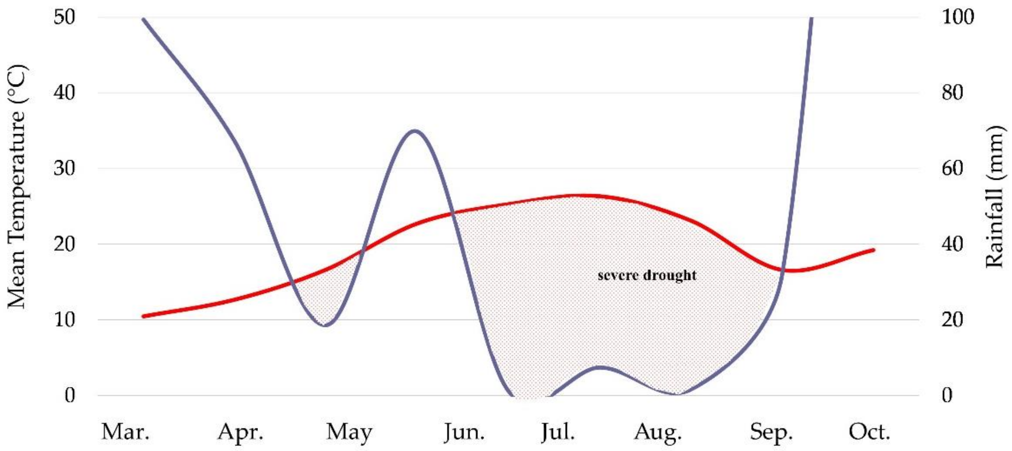

Figure S1. Climatic Bagnouls and Gaussen diagram and dry periods (red area) for the season 2021 in urban vineyard of Botanical Garden of Rome.

Table S1. Two way ANOVA performed to study the effect of berry skin color and as well as ripening period on vegetational indices (MCARI, PRI, CI, BWI, LWI, NDWI) and physiological parameters (QY, gs and CHL) of leaves of 15 selected varieties at BBCH 089-Berries ripe for harvest.

	SS	MS	F	ProbF	Sign.
MCARI					
Skin color	0.01	0.01	0.02	0.88	
Ripening period	5.16	2.58	6.78	0.03	*
Skin color X Ripening period	0.93	0.46	1.22	0.36	
CI					
Skin color	0.73	0.73	1.01	0.35	
Ripening period	2.83	1.41	1.95	0.22	
Skin color X Ripening period	1.75	0.87	1.21	0.36	
PRI					
Skin color	1.16	1.16	1.10	0.33	
Ripening period	1.95	0.98	0.93	0.45	
Skin color X Ripening period	1.51	0.75	0.72	0.53	
BWI					
Skin color	0.33	0.33	0.33	0.59	
Ripening period	1.62	0.81	0.80	0.49	
Skin color X Ripening period	1.41	0.71	0.70	0.53	
NDWI					
Skin color	0.74	0.74	2.54	0.16	
Ripening period	8.86	4.43	15.23	0.00	**
Skin color X Ripening period	0.38	0.19	0.65	0.55	
LWI					
Skin color	3.05	3.05	4.47	0.08	
Ripening period	2.41	1.21	1.77	0.25	
Skin color X Ripening period	0.75	0.37	0.55	0.60	
QY					
Skin color	0.01	0.01	0.01	0.91	
Ripening period	8.44	4.22	5.58	0.04	*
Skin color X Ripening period	0.05	0.02	0.03	0.97	
g_s					
Skin color	0.01	0.01	0.01	0.93	
Ripening period	0.49	0.25	0.24	0.79	
Skin color X Ripening period	5.36	2.68	2.65	0.15	
CHL					
Skin color	0.02	0.02	0.02	0.90	
Ripening period	3.42	1.71	1.84	0.24	
Skin color X Ripening period	2.02	1.01	1.09	0.40	
